# A Review on the Effects of Atrazine on Male Rat Reproductive System Cytoarchitecture, Steroidogenesis and Oxidative Pathway

**DOI:** 10.1007/s10735-025-10671-5

**Published:** 2026-01-13

**Authors:** Elisângela Martins-Santos, Cleida A. Oliveira

**Affiliations:** Department of Morphology, Universidade Federal de Minas Gerais, Av. Presidente Antônio Carlos, 6627, Belo Horizonte, MG 31270-901 Brazil

**Keywords:** Atrazine, Endocrine disruptor, Male reproductive system, Steroidogenesis, Oxidative stress, Prostate cancer risk

## Abstract

Atrazine (2-chloro-4-ethylamino-6-isopropylamino-s-triazine) is an active component of herbicides used worldwide in agriculture. Atrazine is an endocrine disruptor, capable of interfering with the delicate hormonal balance necessary for normal reproductive function. Despite numerous studies on atrazine’s impact on male reproduction, significant gaps remain, particularly concerning the male genital tract beyond the testes. Therefore, we reviewed the effects of atrazine on the entire male reproductive system to understand the complexity of its actions. Adverse effects of atrazine on the morphophysiology of male reproductive organs, including the testes, efferent ductules, epididymis, accessory glands, and external genitalia, all crucial for male fertility, have been found. Atrazine disrupts the hypothalamic-pituitary-gonadal (HPG) axis, affects key enzymes in steroidogenesis, induces oxidative stress, and consequently disrupts spermatogenesis. Moreover, intergenerational effects are evident, with in utero exposure leading to reproductive abnormalities in offspring, and transgenerational effects have also been observed. Effects on rodent prostate and male external genitalia are concerning, considering that higher incidence of prostate cancer, cryptorchidism and hypospadias, potentially related to atrazine exposure have also been described. Given that atrazine is a globally used endocrine disruptor, further studies are required to clarify its potential involvement in these developmental disorders. Future systematic studies using environmentally relevant doses of atrazine, encompassing the entire reproductive system and its control by the HPG axis, are crucial for a thorough risk assessment.

**Graphical abstract:**

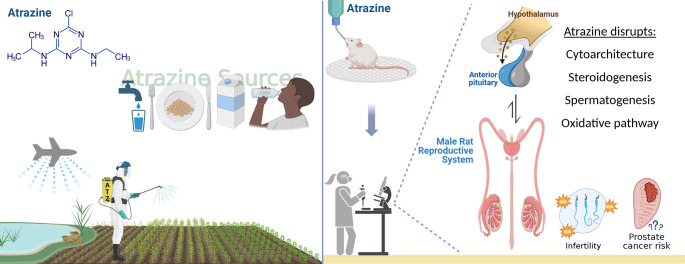

## Introduction

Atrazine (2-chloro-4-ethylamino-6-isopropylamino-s-triazine) is an active component of herbicides widely used in agriculture. It can reach its targets but also distributes to other environmental compartments and negatively affects non-target organisms. As such, the Atrazine low absorption rate, extended half-life in soils, and moderate aqueous solubility led to its widespread detection in agricultural soils (Severi-Aguiar and Silva-Zacarin 2012; Farenhorst et al. 2015; Alonso et al. 2018; Singh et al., 2008; Das et al. 2020; Chen et al. 2024a; Cheng et al. 2024; Mangold et al. 2024). Atrazine may also be detected in the air (Dos Santos et al. 2011; Farenhorst et al. 2015; Das et al. 2020; Amaral Dias et al. 2021; Zhao et al. 2023; Hu et al. 2024; Albaseer et al. 2025), in diverse water sources, including surface waters, groundwater, rainfall, and even drinking water (Nwani et al. 2010; Fairbairn et al. 2016; Elias and Bernot 2017; Alonso et al. 2018; Qu et al. 2020; Owagboriaye et al. 2022; Mangold et al. 2024; Santos et al. 2024; Zhao et al. 2024).

Each country has its own legislation regarding the use of atrazine and sets a Maximum Contaminant Level (MCL) in drinking water or surface water. The World Health Organization (WHO) accepts up to 100 µg/L of atrazine in drinking water (WHO guidelines for drinking- water quality, 2022). In the United States, the U.S. Environmental Protection Agency (EPA) has set a maximum contaminant level for atrazine in drinking water at 3 µg/L. Additionally, atrazine has been considered a restricted-use pesticide, requiring special certification for its application (Agency for Toxic Substances and Disease Registry, 2024). In the European Union, before the ban on atrazine in 2004, the maximum allowable level in surface water and drinking water was 0.1 µg/L. Nonetheless, even after the ban, atrazine has still been found in groundwater in 18 European countries (European Environment Agency 2020). In Canada, a limit of 5 μg/L was established for atrazine in drinking water (Health Canada 1993). In Nigeria, where the herbicide is widely used and where many studies related to its environmental effects have been carried out (Abarikwu et al. 2010; 2011a; 2011b; 2011c; 2012; 2014; 2015; 2020; 2021; 2023; 2024a; 2024b; Aderara et al., 2021; Adesiyan et al. 2011; Farombi et al. 2012; Ndufeiya-Kumasi et al. 2022; Olayinka et al. 2022; Owagboriaye et al. 2022; 2024; Rotimi and Adeyemi 2023; Rotimi et al., 2023), there is no established maximum permissible limit for atrazine in drinking water (Owagboriaye et al. 2024). In Brazil, the legislation allows a maximum level of 2 µg/L of atrazine in drinking water (FIOCRUZ, 2020). However, in the last few years, permissive environmental laws and newly authorized pesticide formulations have been enlarged in the country (Amaral Dias et al. 2021). Accordingly, studies on Brazilian water reservoirs have shown the presence of atrazine at mean levels ranging from 7.0 to 15.0 µg/L (Sousa et al. 2016).

Furthermore, atrazine residues have been identified in everyday food items such as milk and yogurt (Garcia et al. 2012; Li et al. 2013; Song et al. 2014), rice, corn, wheat, and other cereals (Zhang et al. 2014; Zhao et al. 2015). This pervasive contamination poses significant risks to agrochemical workers handling the herbicide, animals inhabiting areas nearby herbicide application sites, and even individuals and animals without direct exposure to the herbicide.

A major concern is that atrazine has been considered a potent endocrine disruptor, capable to cause reproductive disorders in vertebrates (Kniewald et al. 2000; Stoker et al. 2000, 2002; Trentacoste et al. 2001; Ashby et al. 2002; Hayes et al. 2002, 2010, 2011; Tavera-Mendoza et al. 2002; Betancourt et al. 2006; Swan 2006; Suzawa and Ingraham 2008; Rey et al. 2009; Victor-costa et al. 2010; Hussain et al. 2010; Stanko et al. 2010; Tillitt et al. 2010; Papoulias et al. 2014; Richter et al. 2016; Martins-Santos et al. 2017, 2018; Zhu et al. 2021; Chen et al. 2024b; Guimaraes-Ervilha et al. 2024; Oliveira et al. 2024). In this sense, adverse effects of atrazine on male reproductive system have been reported in several species of mammals, including rat, mice, porcine, bovine, marsupials and human, both in vivo and in vitro. These studies were mostly performed at prenatal, early postnatal, peripubertal or pubertal periods, which are critical periods for normal development and growth of the male reproductive organs, thus being susceptible to environmental toxicants.

Exposure of adult male rodents to atrazine has also been extensively studied, revealing a wide range of adverse effects on both non-reproductive and reproductive systems. The testes have been the focus of most research on reproductive effects. Based on evidence from a systematic review and meta-analysis on rodent studies there was consistent and significant damage effect on rodent reproductive organ weight, sperm quality, and serum testosterone levels across different species, life stage, and dosage (Zhu et al. 2021). The mechanism of action of atrazine is still a matter of debate, as disruption in steroidogenesis and/or regulation of the HPG axis, with possible involvement of aromatase, as well as involvement of oxide-inflammatory systems has been proposed. However, a critical knowledge gap still exists regarding the impact of atrazine on extra testicular organs, such as the efferent ductules, epididymis, and accessory glands, as endpoints. These organs play a vital role in sperm maturation, motility, and viability, all essential factors for successful male fertility. Additionally, cancer risk has been pointed as detrimental effects on male reproductive organs, such as testes and prostate (Remigio et al. 2024). Therefore, this review aims to systematically address this gap by integrating both testicular and extra-testicular findings, alongside a detailed analysis of intergenerational and transgenerational effects, to build a more complete picture of atrazine's impact on male reproductive system.

Another point of concern is the context of climate change. Global warming has been projected to impact on the precipitation intensity and storms. This may lead to leaching environmental contaminants, such as atrazine, thus reducing the persistence and efficacy of the herbicide, leading to the need for more applications as well as to increase in groundwater contamination, consequently increasing the risk for human and animal health (Delcour et al. 2015; Barrios et al. 2020; Diagboya and Düring, 2024). Considering the already imposed climate change scenario, associated with the unrestricted use of atrazine in several parts of the globe, it is mandatory to have a more comprehensive understanding of the health effects of this widely used herbicide.

Due to limitations regarding epidemiological evidence on human health effects, the use of rodent, especially rats, as research toxicological model is still needed. This review intends to compile and systematically highlight information regarding the effects of atrazine exposure on the entire male reproductive system, including testicular and extra-testicular targets, with a primary focus on rats. When relevant, findings from other rodents (mice) and translational data (human and in vitro) will be quoted to provide a critical analysis of the mechanisms of action. By systematically highlighting the current known impacts of atrazine on male health and fertility, this review aims to contribute to future decision-making regarding knowledge gaps to be investigated and, ultimately, the herbicide uses and management.

## Atrazine Effects on Testes

Rat atrazine exposure leads to a range of testicular effects, including alterations in testicular weight and morphological changes, ultimately resulting in infertility and irreversible damage. Furthermore, these effects can span generations, even in offspring that were not directly exposed to atrazine.

### Testicular Weight

Significant alterations in testis weight have been reported in male rats exposed to atrazine (Table 1), although with controversial results, as some authors found an increase (Abarikwu et al. 2010; Quignot et al. 2012a; Zhu et al. 2021) or decrease (Pogrmic et al. 2009; Mokhtari et al. 2011; Song et al. 2014; Agdam et al. 2017; Rotimi et al., 2023) in the organ weight, whereas others did not detect a change in testis weight (Kniewald et al. 2000; Stoker et al. 2000; Mokhtari et al. 2011; Farombi et al. 2012; Song et al. 2014). In this sense, Kniewald et al. (2000) did not find any change in adult rat relative testis weight, when treated with atrazine at doses of 60 and 120 mg/kg for 60 days twice a week. Besides the lower dose of the herbicide, these authors used a regimen of just twice a week exposure, which probably was not enough to cause alterations in the testes.

**Table 1:** Effects of atrazine on body and testicular weight

References	Dose (atrazine = ATZ)	Body weight	Testicular weight		Model
			Absolute	Relative	
Stoker et al. (2000)	ATZ 12.5 mg/kg/30d	( =)	( =)	N.A	Prepubertal male Wistar rats (21 to 53 PND)
	ATZ 25 mg/kg/30d	( =)	( =)	N.A	
	ATZ 50 mg/kg/30d	( =)	( =)	N.A	
	ATZ 100 mg/kg/30d	( =)	( =)	N.A	
	ATZ 200 mg/kg/30d	↓	( =)	N.A	
Pogrmic et al. (2009)	ATZ 50 mg/kg/27d	( =)	N.A	↓	Prepubertal male Wistar rats (23 to 50 PND)
	ATZ 200 mg/kg/27d	↓	N.A	↓	
Quignot et al. (2012a)	ATZ 200 mg/kg/14d	↓	↓	↑	Prepubertal male Sprague–Dawley rats (49 PND)
Song et al. (2014)	ATZ 38.5 mg/kg/30d	( =)	( =)	N.A	Prepubertal male Sprague–Dawley rats (28 PND)
	ATZ 77 mg/kg/30d	( =)	( =)	N.A	
	ATZ 154 mg/kg/30d	↓	↓	N.A	
Zhu et al. (2021)	ATZ 120 mg/kg/4 weeks	↓	N.A	↑	Prepubertal male Sprague–Dawley rats (14 PND)
Kniewald et al. (2000)	ATZ 60 mg/kg twice a week for 60 days	↓	( =)	( =)	Adult male Fischer rats
	ATZ 120 mg/kg twice a week for 60 days	↓	( =)	( =)	
Abarikwu et al. (2010)	120 mg/kg/7d	( =)	( =)	**↑**	Adult male Wistar rats
	200 mg/kg/7d	↓	( =)	**↑**	
	120 mg/kg/16d	( =)	( =)	**↑**	
	200 mg/kg/16d	↓	( =)	**↑**	
Victor-Costa et al. (2010)	ATZ 300 mg/kg/7d	↓	**↑**	**↑**	Adult male Wistar rats
	ATZ 50 mg/kg/15d	( =)	( =)	( =)	
	ATZ 200 mg/kg/15d	↓	**↑**	**↑**	
	ATZ 200 mg/kg/40d	↓	↓	↓	
Mokhtari et al. (2011)	ATZ 100 mg/kg/14d	↓	( =)	N.A	Adult male Wistar rats
	ATZ 200 mg/kg/14d	↓	↓	N.A	
	ATZ 400 mg/kg/14d	↓	↓	N.A	
Farombi et al. (2012)	ATZ 120 mg/kg/16d	( =)	( =)	( =)	Adult male Wistar rats
Agdam et al. (2017)	ATZ 200 mg/kg/48d	( =)	N.A	↓	Adult male Wistar rats
Martins-Santos et al. (2017)	ATZ 200 mg/kg/7d	( =)	( =)	↑	Adult male Wistar rats
	ATZ 200 mg/kg/14d	( =)	( =)	↑	
	ATZ 200 mg/kg/40d	↓	( =)	↑	
	ATZ 200 mg/kg/40d + 75d rec	( =)	↓	↓	
Rotimi et al., (2023)	ATZ 120 mg/kg/7d	( =)	↓	( =)	Adult male Wistar rats

↑ increase, ↓ decrease, ( =) Not Changed, N.A: Not Assessed

An increase in relative testicular weight was found by Abarikwu et al. (2010) and Quignot et al. (2012a), who used similar dose and time of treatment (200 mg/kg for 7 to 16 days and 14 days, respectively). On the other hand, even using the same dosage of atrazine (200 mg/kg), Pogrmic et al. (2009) and Agdam et al. (2017), found a reduction in testicular weight, after a longer period of exposure (for 27 and 48 days, respectively). Rotimi et al. (2023) reported a decrease in absolute testicular weight in male Wistar rats following administration of a 120 mg/kg dosage of atrazine for 7 days, but the organ weight was similar to the control when calculated as relative to body weight. According to Abarikwu et al. (2010), the absolute testicular weight remained unaffected at doses of 120 and 200 mg/kg, but when expressed relative to body weights, the testicular weight was significantly increased. On the other hand, Song et al. (2014) found a reduction in testicular weight following atrazine exposure at a dose of 154 mg/kg for 30 days; however, they did not find changes in testicular weight at lower doses during the same period (38.5 and 77 mg/kg for 30 days). Higher doses of atrazine (200 mg/kg and 400 mg/kg) were also needed to decrease adult rat relative testicular weight, following peritoneal injection of atrazine for 14 days, whereas 100 mg/kg had no effects on this parameter (Mokhtari et al. 2011).

It is important to highlight that the conflicting results regarding testicular weight appear largely dependent on the specific exposure protocol (dose and duration), but not on rat strains (Wistar, Sprague–Dawley or Fisher rats), and the developmental window at which the effect was assessed (adult and pre/peripubertal). In general, doses of 100 mg/kg of atrazine or below do not affect the adult testis weight, whereas the weight varied greatly at 120 mg/kg atrazine or above, with increase, decrease or no changes detected even in similar periods of exposure.

Results from previous studies of our research group showed that up to a 50 mg/kg dose, atrazine did not alter the testis weights after 15 days of exposure (Victor-Costa et al. 2010), however higher doses of the herbicide (200 mg/kg for 15 days and 300 mg/kg for 7 days) led to an increase in testis weight, whereas drastic decrease in the weight was observed following a longer period of treatment (200 mg/kg for 40 days) (Victor-Costa et al. 2010). Martins-Santos et al. (2017) also found an increase in relative testicular weight even at shorter exposure to atrazine (200 mg/kg for 7 and 14 days). On the other hand, a reduction in testes weight was also found to correlate with drastic testicular atrophy, characterized by mostly Sertoli-cell-only seminiferous tubules after longer treatment (Victor-Costa et al. 2010). These changes, depending on dose and length of treatment, suggested a way to reconcile the previous divergent reports concerning testicular weight, as the increase in weight correlated with testicular swelling, characterized by seminiferous tubule luminal dilation, possibly resulting from fluid accumulation, and the decrease in testis weight correlated with testicular atrophy (Victor-Costa et al. 2010).

Testicular swelling followed by atrophy of the seminiferous tubules is a common finding for several toxicants that affect the male reproductive tract, especially the efferent ductules (Nakai et al. 1992, 1993; Hess et al. 1997; Hess and Cooke 2018). In fact, we found that the increase in testicular weight observed at high doses and long exposure to atrazine may indeed result from a failure in the efferent ductules to reabsorb luminal fluids (Martins-Santos et al. 2018), as discussed in the section regarding efferent ductules. It has been proven that the excess of luminal fluid returns to the testes, initially causing an increase in testicular weight due to fluid accumulation, followed by testicular atrophy and a consequent reduction in the testis weight (Hess et al. 1997; Oliveira et al. 2001; 2002; Martins-Santos et al. 2018).

These data highlight the importance of examining multiple timepoints, in order to better assess the extent of the herbicide effects in the testis, as changes in the organ weight and structure may be associated with other factor, such as post testicular effects, body weight (not always take in account in some studies), and treatment regimen, as dose and length of the exposure.

### Testicular Morphology

Parallel to alterations in testicular weight, the changes on testicular morphology were also heterogeneous, as normal and abnormal seminiferous tubules were co-localized in the same sections, indicating that morphological alterations occurred gradually (Victor-Costa et al. 2010; Martins-Santos et al. 2017; 2018; Abdel Aziz et al. 2018). Morphological alterations in the rat testes, induced by atrazine, were first described by Kniewald et al. (2000), who reported a small number of spermatogenic cells per unit of area, along with spermatogenic cells exhibiting signs of death and disorganized seminiferous tubules. Others have reported testicular degeneration, including reduction in tubular diameter and seminiferous epithelium thickness (Ndufeiya-Kumasi et al. 2022; Mgbudom-Okah et al. 2023), presence of luminal sloughed cells in seminiferous tubules (Abarikwu et al. 2010), seminiferous epithelium degeneration and reduced sperm cells (Adedara et al. 2021; Ndufeiya-Kumasi et al. 2022; Ikeji et al. 2023), as well as sperm maturation arrest in atrazine-treated rats, which presented testicular tissue mostly composed by early-stage seminiferous tubules containing spermatocytes and only a few maturing spermatids (Rotimi et al., 2023).

Other severe testicular damages in rats exposed to atrazine have also been described (Agdam et al. 2017; Abdel Aziz et al. 2018; Hassanin et al. 2024). The damages were manifested as disorganized seminiferous tubules filled with abnormal and degenerating germ cells, necrotized and vacuolized cells, some tubules presenting only one or two germ cell layers (Abdel Aziz et al. 2018), as well as seminiferous tubules shrunken with irregular outlines atrophy, detached germinal epithelium, cells shed and sloughed to the lumen, and few or absent sperm (Agdam et al. 2017; Hassanin et al. 2024). Disruption in the development of the testes structure, including destructions of the seminiferous tubules, multinucleated cells shed in the lumen, vacuoles in the seminiferous epithelium, and reduced and disordered spermatogenesis have also been registered (Zhu et al. 2021). In line with these findings, a more recent study also showed that atrazine induced alterations in testicular morphology, coupled with increased germ cell apoptosis and fibrosis, resulting in decreased spermatogenesis and impaired male fertility in adult rats (Hassanin et al. 2024).

At the ultrastructural level, degenerative changes were found in Sertoli cells, as the nucleus was irregular in shape and the cytoplasm presented large and irregular lysosomes as well as numerous vacuoles, especially at the adluminal surface, where degenerated spermatids were also visualized (Kniewald et al. 2000). Altered Sertoli cells with vacuolations, dilated smooth endoplasmic reticulum, vacuolated mitochondria, lipid droplets and multivesicular bodies in the cytoplasm, as well as abnormal blood vessels and significantly damaged interstitial tissue, with reduced Leydig cell has also been reported after atrazine exposure (Agdam et al. 2017; Hassanin et al. 2024).

Reduction in Leydig cell population has been found in a dose- and time-dependent exposure to atrazine (Feyzi-Dehkhargani et al. 2012; Agdam et al. 2017). Morphology of Leydig cells after atrazine exposure appears irregular in shape, reduced in size and contained enlarged rough endoplasmic reticulum, several lysosomes and cytoplasmic vacuoles (Kniewald et al. 2000), as well as hypertrophied with granulated cytoplasm, mostly presenting faint lipophilic cytoplasm (Agdam et al. 2017). Decrease in Leydig cell number and hypertrophy of the remaining cells has also been described (Feyzi-Dehkhargani et al. 2012). Adding to these observations from histological data, Victor-Costa et al. (2010) reported alterations at ultrastructural level, such as pleomorphic Leydig cells, usually smaller than those in the control group and exhibiting irregular nucleus with alterations consistent with cell death.

Substantiating the adverse effects of atrazine in the rat testes, as evidenced by the significant reduction in germ cells, Sertoli cells, and Leydig cells count, Mgbudom-Okah et al. (2023) investigated specifically the Sertoli and Leydig cells as target, by using Leydig cell line (TM3) and Sertoli cell line (TM4), respectively. The herbicide decreased the viability of TM3 Leydig cells, but not the TM4 Sertoli cells, and induced reactive oxygen species (ROS) production in both TM3 and TM4 cell lines. Coadministration of testosterone with atrazine reverted the testes and cell lines herbicide effects, thus suggesting that the toxicity occurred by oxidative stress and could be reversed by testosterone (Mgbudom-Okah et al. 2023). Furthermore, atrazine has been shown to directly target Leydig cells, leading to their dysfunction and death. For instance, exposing primary cultures of rat Leydig cells to high atrazine concentrations (25 and 50 μg/mL for 48 and 72 h) significantly decreased cell viability, thus demonstrating a direct effect of atrazine on these cells (Abarikwu et al. 2011a).

Corroborating data from the literature, we found profound alterations in testes of rats at short-term exposure and high doses of atrazine (200 mg/kg for 15 days and 300 mg/kg for 7 days), which resulted in dilation of the seminiferous tubules, whereas long-term exposure (200 mg/kg for 40 days) led to testicular atrophy. The testicular atrophy was characterized by reduction in the number of germ cells with the presence of large, multinucleated bodies, and abundant apoptotic cells within the tubules indicating cell death and a dysfunctional seminiferous epithelium, resulting mainly in Sertoli-only tubules (Victor-Costa et al. 2010; Martins-Santos et al. 2017). Noteworthy, our data was the first to show that the alterations promoted by chronic exposure to atrazine (200 mg/Kg for 40 days) are not reversible after long period (75 days) of cessation of atrazine treatment, as the testes were significantly reduced in size, and presented seminiferous tubules containing Sertoli-only cells or, eventually, Sertoli Cells and rare spermatogonia. This recovery time is sufficient for covering more than one spermatogenic cycle (58 days) in the rats (Russel et al. 1990). Within this timeframe studied, the formation of spermatocytes was not observed, despite occasional signs of spermatogonia mitosis in few atrophic tubules, thus suggesting that, although there was an attempt to recover spermatogenesis, the effects of atrazine on spermatogenesis could not be reverted (Martins-Santos et al. 2017).

One major criticism of most studies on the effects of atrazine on animal reproduction is that the dosages used represent supra-environmental exposure, thus becoming rather unlikely to have practical relevance. Nevertheless, a recent study by Riera et al. (2022) found damages in rat testicular tissue, characterized by a dramatic decrease in the germinal epithelium, and the absence of sperm, despite having used lower doses of atrazine (13 μg/L up to 50 μg/L). The authors highlighted that 13 μg/L atrazine was the concentration previously found in the La Villa River (Province of Los Santos, Panamá), in 2015, thus evidencing the potential health risk for the population. Similarly, Owagboriaye et al. (2022) reported mild rat testicular degeneration after exposure to atrazine concentrations found in drinking water at several Nigeria locations (0.08 mg/L), further emphasizing the risk of environmentally relevant dosages of atrazine.

Altogether, the structural alterations of the testes may explain the significant reduction in daily sperm production and sperm viability in rats (Abarikwu et al. 2010; 2014; 2020; Farombi et al. 2012; Aderara et al., 2021; Pandey et al. 2021; Rotimi et al. 2024), highlighting a potential risk of infertility in animals exposed to atrazine. Atrazine has also been associated with poor semen quality across species, including humans, bovines (Komsky-Elbaz, 2017, 2019; Roth et al. 2020), pigs (Betancourt et al. 2006), and rodents (Kniewald et al. 2000). The herbicide reduced total testicular sperm count, as well as epididymal and seminal sperm count, motility, and vigor (Kniewald et al. 2000; Tavera-Mendoza et al. 2002; Swan et al. 2003; Betancourt et al. 2006; Abarikwu et al. 2010; Gely-Pernot et al. 2015; Komsky-Elbaz, 2017, 2019; Kale et al. 2018; Aderara et al., 2021; Owagboriaye et al. 2022; Rotimi et al. 2024). Corroborating these findings, exposure to atrazine has been linked to a high number of abnormal and dead sperm cells (Abarikwu et al. 2010, 2020; Farombi et al. 2012; Feyzi-Dehkhargani et al. 2012; Abdel Aziz et al. 2018; Kale et al. 2018; Aderara et al., 2021; Ikeji et al., 2023; Rotimi et al., 2023). Together, these data reflect the effects of atrazine on testes but do not exclude possible effects downstream of the testes for guaranteed production of good-quality semen.

### Intergenerational and Transgenerational Effects of Atrazine

In addition to effects of atrazine on adult male rats, it has been shown that in utero exposure also disrupted rat fetal testes development and caused damage in the reproductive system affecting male offspring of the F1 and F2 generations (Pandey et al. 2021). Furthermore, effects extending to the F3 generation have also been established (McBirney et al. 2017; Thorson et al. 2020; Beck et al. 2022; He et al. 2022), demonstrating the transgenerational potential of the compound.

Exposure to atrazine during pregnancy and lactation caused delayed testicular descent, sperm count, and motility, along with testicular defects and subfertility in male offspring (F1 generation), as well as morphological deformities in F2 fetuses (Pandey et al. 2021), highlighting the intergenerational effects of atrazine. The adverse effects detected by Pandey et al. (2021) occurred in parallel to decrease in serum testosterone, but increase in serum estrogen levels, as well as upregulation of testicular expression of androgen receptor (AR), estrogen receptors (ERα and ERβ), steroidogenic acute regulatory protein (StAR), aromatase, and Insulin-like 3 (INSL-3), thus indicating estrogenic and anti-androgenic activity of atrazine.

Similarly, the upregulation of *INSL-3* gene following gestational exposure to atrazine (25–100 mg/kg) has been associated with disrupted fetal testis development and impaired Leydig or Sertoli cell function (Fang et al. 2018). Moreover, Atrazine exposure resulted in reduced serum testosterone levels in male pups, indicating interference with androgen production, besides increase in mitosis of fetal Leydig cells and abnormal aggregation of these cells. Despite the increase in Leydig cell number, expression of key genes (*Scarb1, Cyp17a1*) involved in androgen biosynthesis was downregulated by atrazine. Atrazine also down-regulated *Hsd17b3*, a crucial enzyme for testosterone production, but upregulated *Sox9* and *Fshr*, without affecting normal Sertoli cell lineage development. Thus, atrazine disrupts the normal development and function of the fetal testes, leading to long-term reproductive disorders (Fang et al. 2018).

Besides rats, multi-generational effects of chronic exposure of mice to an environmental concentration of atrazine (0.02 ng/mL, measured in Australian waterways), also revealed alteration in testicular steroidogenic gene, including downregulation of *Cyp17a1* and *Ddx4* (a germ cell marker) and upregulation of *Star,* even though without impact on expression of *Hsd3b1* (Leydig cell marker) and *Gata6* (Sertoli cell marker), also affecting testicular gross morphology and germ cell parameters, such as sperm motility (Kolaitis et al. 2023).

Another study using marsupial tammar wallaby as animal model for investigating direct effect of atrazine on the developing testis, pointed out that, by interfering with the balance of androgen and estrogen, atrazine disorganized laminin formation during the male pouch programming window, thus resulting in reduced number of germ cells and Sertoli cells (Chen et al. 2024b). These authors also found downregulated *SRY* gene expression and impaired SOX9 nuclear translocation in animals treated with atrazine, further corroborating that atrazine may compromise testis differentiation.

Furthermore, the transgenerational inheritance of Atrazine-induced disease (observed in the F3 generation) is mechanistically explained by epigenetic modifications in the male germline. Studies have demonstrated that in utero exposure to Atrazine in F0 dams leads to unique sets of sperm epimutations in the unexposed F3 generation (McBirney et al. 2017; Thorson et al. 2020; Beck et al. 2022). These epimutations include widespread alterations in DNA methylation (Differential DNA Methylation Regions or DMRs) and abnormal Histone Retention (Differential Histone Retention Regions or DHRs) in the F3 sperm. These specific germline epigenetic biomarkers are correlated with the later development of diseases, such as kidney, prostate, and testicular pathologies, in the F3 and subsequent generations, thereby establishing Atrazine as an epigenetic transgenerational toxicant.

## Atrazine Effects on Male Rat Reproductive Tract

The atrazine effects on extra testicular organs of the male genital system have been much less investigated than testes. This is a regrettable caveat in our understanding of the risk of atrazine on reproduction, considering that, even though the sperm are produced in the testes, the efferent ductules, epididymis and accessory glands are also essential for maintaining the male fertility.

### Efferent Ductules

The efferent ductules connect the rete testis to the epididymis, and they play an important role in the reabsorption of fluid coming from the testes, a process finely regulated by estrogens (Hess et al. 1997, 2000, 2011, 2018, 2005; Lee et al. 2001; Oliveira et al. 2001; 2002; Zhou et al. 2001). It is now well known that disruption in the balance of androgen and estrogen levels in the efferent ductules may result in male infertility, primarily due to luminal fluid accumulation and posterior backflow to the testis, thus resulting in luminal dilation of the seminiferous tubules followed by atrophy and, consequently, infertility (Hess, et al. 1997; Mckinnell et al. 2001; Oliveira et al. 2001; 2002; Cho et al. 2003; Hess and Cooke 2018).

**Fig. 1:**
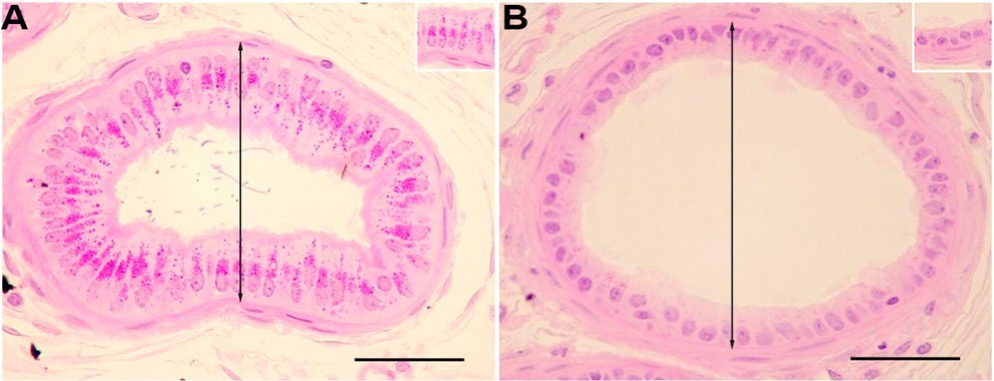
Effects of atrazine on the morphology of adult rat efferent ductules. A = control; B = atrazine treated at 200 mg/kg/40 days; The double arrow indicates luminal diameter. Bars = 100 µm. Insert in A: detail of normal epithelium; Insert in B: detail of atrophied epithelium

Despite the proven importance of the efferent ductules for male reproduction, this segment has been mostly neglected in toxicity studies. Indeed, regarding exposure to atrazine, the only data available regarding the effect on the efferent ductules originates from our research group (Martins-Santos et al. 2018). We have demonstrated that atrazine caused remarkable alterations in these ductules, including luminal dilation, reduction in epithelial height (Fig. 1), and imbalance between proliferation and apoptosis in the epithelial cells. The efferent ductules luminal dilation increased significantly from day 7 to day 15 and 40 of the treatment but returned to the control level after the recovery period. The changes in the efferent ductules were observed paralleling increased aromatase expression in the epithelium (Martins-Santos et al. 2018).

Increased aromatase, the enzyme that converts testosterone to estrogens, has long been described as a consistent effect of atrazine in several organs and animal models (Crain et al. 1997; Sanderson et al. 2000, 2001; 2002, 2006; Hayes et al. 2002; 2006; Heneweer et al. 2004; Laville et al. 2006; Fan et al. 2007a, 2007b; Holloway et al. 2008; Tinfo et al. 2011; Quignot et al. 2012b; Jin et al. 2013; Hudon Thibeault et al., 2014; Caron-Beaudoin et al. 2016).

Interestingly, the increase in aromatase expression in the efferent ductules appeared earlier (7 days) and persisted longer (even after 75 days recovery) compared to the testes, where increase in aromatase occurred only after 40 days of rat exposure (Martins-Santos et al. 2017, 2018). Also noteworthy, the effects on efferent ductules luminal dilation preceded those observed in the seminiferous tubules. As such, taken together, these findings emphasize that the efferent ductules may be a target of atrazine, and at least part of the effects of this herbicide on the morphophysiology of the testes (transient weight increase, seminiferous tubules luminal dilation, followed by drastic atrophy) may be secondary to changes in the efferent ductules (Martins-Santos et al. 2018).

These atrazine effects on efferent ductules and testes mirrored those seen in animal models with genetically or chemically disrupted balance on androgen and estrogen levels (Hess et al. 1997; Mckinnell et al. 2001; Oliveira et al. 2001, 2002; Cho et al. 2003), as well as those promoted by exposure to other environmental toxicants and therapeutic compounds (Nakai et al. 1992, 1993; Hess 1998, 2014; Gotoh et al. 1999; La et al. 2012). The mechanism involved in the luminal dilation of the efferent ductules promoted by disruption in the androgen/estrogen balance appears to be related to compromised epithelial structure and fluid reabsorption pathways, as shown by decreased NHE-3, aquaporins 1 and 9 (AQP1, AQP9), fibrosis transmembrane conductance regulator (CFTR), as well as Na + /K + ATPase (Oliveira et al. 2002; 2005; Lee et al. 2001; Zhou et al. 2001; Hess 2014). For other toxicants, such as the fungicide Benomyl and its metabolite Carbendazim, as well chemical drug, impairment of fluid reabsorption and backflow to the testis is mostly related to occlusion of efferent ductules by sloughed germ cells and cellular debris, or even by granulomatous inflammation (Nakai et al. 1992; 1993; Gotoh et al. 1999; Hess and Nakai 2000; La et al. 2012; Hess 2014). Even though the mechanisms of testicular atrophy may be diverse, there are convincing evidence that efferent ductules may take part in this process, thus indicating that attention to this male tract segment should be made before conclusions on long-term outcome on testicular structure as a primary target for toxicity.

Further emphasizing the effects of atrazine in the rat efferent ductules, significant increase in epithelial cell proliferation but not apoptosis, especially in the distal portion of this segment, was also found, paralleling the effects on aromatase expression (Martins-Santos et al. 2018). It has been proposed that aromatase induction may increase the local estrogen level, thus influencing cell proliferation, possibly to compensate the luminal dilation observed in the ductules. Interestingly, the epithelial proliferation returned to control levels after atrazine discontinuation (recovery group), coinciding with cessation of the tubule’s dilation.

Studies regarding the physiological regulation of cell proliferation and death on the efferent ductules are scarce or even lacking, especially considering the effects of atrazine, thus making difficult any comparison. However, it was interesting to find that another study on the efferent ductules of the neotropical bat *Artibeus lituratus* also found that the estrogen responsive system is a key factor determining the regulation of the efferent ductules tissue homeostasis, as changes in cell proliferation but not death positively correlated with the expression of estrogen receptor alpha (ERα), a known proliferation factor in several cellular systems (Campolina-Silva et al., 2019).

### Epididymis

Effects of atrazine exposure on the epididymis included alterations in the organ weight, which has been shown to be decreased in rats exposed to the herbicide (120 mg/kg for 7 days—Rotimi et al., 2023; 200 mg/kg for 30 days—Stoker et al. 2000, 2002; 200 mg/kg for 7 and 16 days—Abarikwu et al. 2010). Decrease in the epididymis weight was also found in rat offspring exposed to atrazine through gestation and lactation period (2, 10, 70 and 100 mg/kg in F0 dams from gestational day 6 to postnatal day 21) (Pandey et al. 2021). No changes in relative epididymis weight were observed in rats treated with lower dosages of atrazine (60 and 120 mg/kg for 60 days twice a week—Kniewald et al. 2000; 120 mg/kg for 7 and 16 days—Abarikwu et al. 2010; 38.5, 77 and 154 mg/kg for 30 days—Song et al. 2014). Therefore, the dose regimen and time of exposure appear to be mandatory for affecting this male tract segment.

Considering epididymal morphology, previous studies by Stoker et al. (2002) and Abarikwu et al. (2010) did not observe histological changes after exposure to atrazine. However, degeneration of the epididymis has been described, with decreased tubular diameter, epithelial vacuolation, irregular lumens, scarcity or absence of sperm within the lumen, and increased luminal sperm cell abnormalities and degeneration (Abdel Aziz et al. 2018; Adedara et al. 2021; Ikeji et al. 2023). Rotimi et al. (2023) also reported that the epididymal tissue of atrazine-treated rats had convoluted ducts lined by cuboidal epithelium, containing significantly reduced density of spermatozoa.

**Fig. 2:**
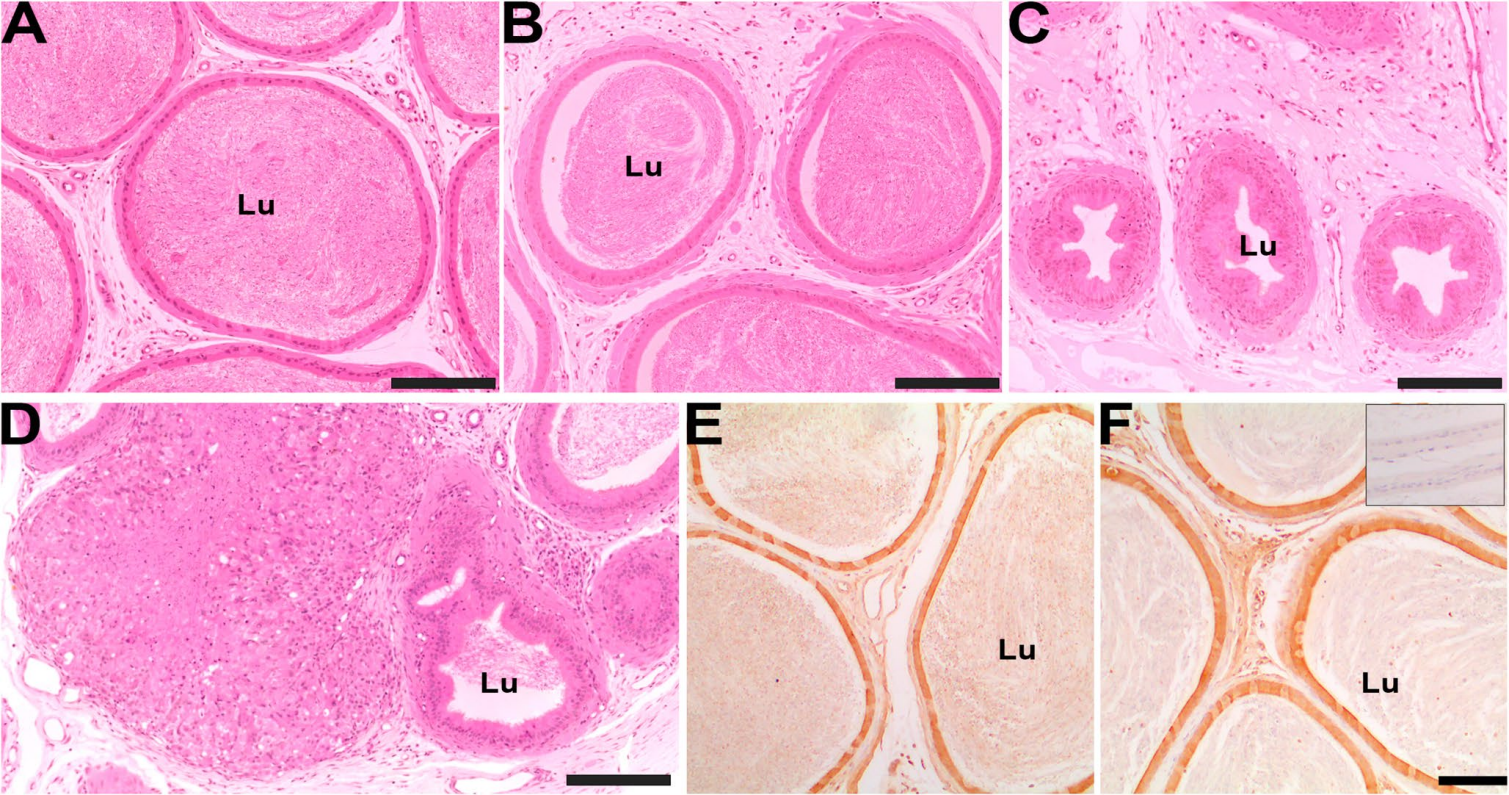
Effects of atrazine on the morphology of the adult rat cauda of the epididymis (EP). A and E = control; B – D and F = atrazine treated at 200 mg/Kg/40days; B = treated EP showing normal-appearing ducts; C = evidence of ductal atrophy; D = EP granuloma; E and F = Immunolocalization of aromatase. Insert in F: negative control. Bars in A - D = 200 µm; Bar in F = 100 µm; Lu = Lumen

Expanding previous information, by using the same experimental animals used for efferent ductules description (for methodology details see Martins-Santos et al. 2017, 2018), we showed that exposure to atrazine (200 mg/Kg/40 days followed or not of a recovery period of 75 days) promoted heterogeneous morphological changes in the epididymis, as some ducts were normal in appearance, whereas others exhibited alterations such as reduced lumen size, shrinkage, reduced intraluminal sperm (sometimes progressing to azoospermia), focus of inflammation and occasional granulomas, ultimately leading to ductal obstruction and degeneration (Fig. 2). Following the recovery period, the morphological changes were more pronounced, with persistent inflammatory infiltrates, and azoospermia.

Alteration in tissue homeostasis was also observed, as exemplified by increased cell proliferation (63%), apoptotic figures and caspase-3 positive cells (240%) in the head of the epididymis after 40 days of chronic atrazine treatment (unpublished data). All studies conducted so far are consistent in indicating that atrazine induces the process of cell death by apoptosis, however for the epididymis our data is unprecedented.

The mechanism underlying the changes in the epididymis is still not well defined, even though alterations in oxide-inflammatory systems, with reduced Glutathione—GSH and antioxidant enzymes, such as Catalase—CAT, Superoxide Dismutase—SOD, and Glutathione S-transferase—GST (Ikeji et al., 2023; Rotimi et al., 2023), and increased oxidative stress, as seen by the Malondialdehyde—MDA levels (Rotimi et al., 2023), as well as increase in inflammation markers, including nitric oxide—NO and myeloperoxidase—MPO (Ikeji et al., 2023; Rotimi et al., 2023) have been detected in the tissue after exposure to atrazine.

Directly impacting epididymal function, it has been demonstrated that biochemical function indices decreased significantly after atrazine exposure, leading to a consequent decrease in semen quality parameters in the rat epididymis (Pandey et al. 2021; Rotimi et al., 2023). This functional decline is determined by decreased sperm concentration, motility, viability and increase in sperm abnormalities in the cauda epididymis (Abarikwu et al. 2010, 2014; Adedara et al. 2021; Ikeji et al., 2023; Rotimi et al., 2023). Considering that the luminal sperm comes from testis and efferent ductules, which are greatly impacted by atrazine, it is possible that the impact on sperm quality is.

## Atrazine Effects on Male Rat Accessory Glands

Studies related to the effects of atrazine on the male reproductive accessory glands remain scarce, despite though reduction in ventral prostate and seminal vesicle weights, that parallel decreased testosterone levels have long been reported (Kniewald et al. 2000; Stoker et al. 2000, 2002; Trentacoste et al. 2001; Pogrmic et al. 2009; Abarikwu et al. 2010). Farombi et al. (2013) also observed a reduction in relative prostate weight, but the authors did not specify the prostate lobe in their study, hindering the interpretation of the results. One possible explanation for the effects on prostate weight may be the reduction in local conversion of testosterone to dyhydrotestosterone (DHT) (Kniewald et al. 1995), which is the main androgen responsible for maintenance of the gland structure and function. This inference is based on previous studies showing reduction in 5α-reductase activity, both in vivo and in vitro, not only in prostate but also in anterior pituitary tissue (Kniewald et al. 1979, 1995, 2000). Concurrently, it has been shown that the number of binding sites for DHT as well as formation of DHT and androgen receptor complex is also decreased in the prostate tissue (Kniewald et al. 1995).

However, some discrepancy in the results regarding prostate weight has also been found. In this sense, Stoker et al. (1999) observed an increase in the absolute weight of the ventral prostate in young Wistar rats (120 days of age) at a dose of 6.25 mg/kg, even though the relative prostate weight was not significantly different, and the weight of the lateral prostate remained unaltered with the treatment. Song et al. 2014 did not find a significant difference in prostate weight after atrazine exposure. Lack of effects on the ventral prostate and seminal vesicle weights has also been described in offspring of dams (pnd 60) exposed to atrazine, although intratesticular and serum testosterone levels were decreased (Rosenberg et al. 2008).

In contrast to ventral prostate, two different research groups showed enlarged lateral prostate at mature age, by using male exposed prenatally to atrazine as model (Stoker et al. 1999; Rayner et al. 2007). The lateral prostates of these animals were characterized by multiple foci of inflammation, mainly composed of neutrophils, present in the gland lumen, as well as mononuclear cells (lymphocytes, macrophages and plasma cells), present in the stroma (Stoker et al. 1999; Rayner et al. 2007). Mild stromal fibrose was also reported (Stoker et al. 1999). The effects on the lateral prostate were more severe when atrazine exposure was extended to lactational period (Rayner et al. 2007).

Chronic prostatitis with predominantly lymphoid infiltration, diffuse and extensive interstitial inflammation affecting more than 75% of the gland, often accompanied by interstitial fibrosis was also found in male rats exposed prenatally to atrazine (Stanko et al. 2010). Moreover, McBirney et al. (2017) found atrophic or hyperplastic prostate glandular epithelium in the F1, F2, or F3 atrazine lineage generations.

Using experimental model of adult male rats exposed to atrazine for 7, 15, and 40 days, we found mild alterations on ventral prostate (Martins-Santos et al. 2018). The structure of the ventral prostate showed significant variation between the animals exposed to atrazine and across treatment periods. This variation included glands with a normal appearance and others containing cystic or hyperplasic acini. Additionally, there were frequent inflammatory cells in the stroma, especially after the longest treatment period (40 days). These inflammatory cells persisted even after the recovery period (75 days after the treatment). Interestingly, aromatase expression in the ventral prostate remained unchanged despite atrazine exposure, indicating that atrazine effects on this enzyme were limited to other reproductive tissues, such as the efferent ductules and testes (Martins-Santos et al. 2017, 2018). Also differing from the efferent ductules, cell proliferation in the ventral prostate was diminished after atrazine exposure but returned to control level in the recovery animals (Martins-Santos et al. 2018). These results further corroborate the tissue specific responses to the herbicide.

In contrast to findings in prostate tissue from animals directly exposed to atrazine, studies using prostate cell lines (RM1 cells, which are androgen independent) indicate that atrazine can stimulate prostate cancer cell growth, progression through the cell cycle, and invasive behavior (Hu et al. 2016). In the same study, xenograft of RM1 cell was generated to evaluate the in vivo effects of atrazine, thus evidencing that the herbicide accelerated the growth of tumor cells in a dose-dependent manner. Another study using human LNCaP prostate cells (which express functional AR but do not express ERα) found no evidence of cell proliferation and PSA secretion following atrazine exposure (Robitaille et al. 2015), thus emphasizing that the herbicide did not exert antiandrogenic effects in LNCaP cells.

Regarding human, studies exploring the relationship between atrazine and prostate cancer in agricultural workers have been controversial (Mills 1998, 2003; MacLennan et al. 2002, 2003; Hessel et al. 2004; Sathiakumar et al. 2011). One analysis of pesticide uses and cancer incidence rates in California counties found a significant correlation between the amount of atrazine applied annually per county and cancer incidence for black males (Mills 1998). Later, another study analysis on cancer incidence at a plant producing atrazine herbicide in Louisiana-USA, from 1985 through 1997, raised questions regarding risk of prostate cancer on men under 60 years of age (MacLennan et al. 2002), however, without evidence of associated cause-specific mortality (MacLennan et al. 2003). The study was considered limited by its small size, by the relatively young age and short follow-up of its subjects, and by the lack of exposure data to support a causal relation between atrazine and prostate cancer (MacLennan et al. 2003).

In 2014, an updated analysis of cancer incidence in a cohort of farm workers in California, USA, from 1988 to 2010, found that these farm workers experienced proportionally more prostate cancer than reference populations (Mills et al., 2014). The authors found that specifically for prostate cancer the incidence increased from 167 to 749, compared to previous follow-up through 1987–1997 (Mills 1998). They assume that the additional follow-up has allowed a longer latency period between potential exposures and cancer occurrence (Mills et al., 2014).

Recently, Remigio et al. (2024) updated the epidemiological 2011 analysis of atrazine within the Agricultural Health Study (AHS) cohort, by incorporating an additional 8.5 average years of follow-up on incidence of cancer at multiple sites and they also found associations of the herbicide with total and aggressive prostate cancer among those applicators diagnosed at younger ages (below 60 years old). Although these findings provide evidence of potential associations between occupational atrazine use and cancer risk (Remigio et al. 2024), further research is still needed to confirm this association.

The inconsistencies observed across these human studies largely stem from well-known methodological challenges inherent to environmental epidemiology, including variations in exposure assessment methodology, insufficient control for complex occupational and lifestyle confounders, and the critical requirement for adequate latency periods in long-term cancer risk evaluations.

## Effects of Atrazine on the Male External Genitalia

The effects of atrazine on male external genitalia malformation, such as hypospadias and cryptorchidism, have been poorly explored in rats, so for this description we will include other species, besides rats.

Prenatal atrazine exposure induced alterations in mice penile morphology, including reduction in glans length and penis size, abnormal Male Urogenital Mating Protuberance (MUMP) morphology, as well as prevention of urethral closure, leading to hypospadias, and descent of testis, leading to cryptorchidism in the offspring (Govers et al., 2020; Tan et al. 2021). Incidence of hypospadias in 10.23% offspring has also been reported after exposure of pregnant rats to 200 mg/kg/d atrazine (Wu et al. 2007).

The potential molecular mechanisms underlying the mice prenatal atrazine-induced developmental defects appear to rely on disruption of developmental gene networks, such as those related to testosterone production (*Star, Cyp17a1, Hsd17b4*), thus decreasing androgen action, as well as other genes related to penile development and masculinization, such as the peptide hormone insulin-like 3 (*Insl-3*) (Tan et al. 2021). Pandey et al. (2021) also found elevated *Insl-3* expression and delayed testis descent in F1 male rats, indicating an anti-androgenic effect of atrazine.

Based on a case–control study, maternal residential atrazine exposure during pregnancy have also been linked to hypospadias, micropenis and cryptorchidism in human populations as well (Agopian et al. 2013). In contrast, Winston et al. (2016) found a weak or no association between hypospadias and daily maternal atrazine exposure via drinking water, during the critical window of genitourinary development.

As the incidence of cryptorchidism and hypospadias in humans has been increasing over time, further studies are still needed to clarify the overall effects of atrazine as an endocrine disruptor on male external genitalia, including in men.

### Translational Relevance and Limitations of Dosage

Although we have discussed the translational relevance of atrazine for human disorders such as hypospadias, cryptorchidism, and prostate cancer, it is essential to contextualize the exposure levels used in the literature. Most studies in animal models utilize doses ranging from 5 to 200 mg/kg of atrazine. These doses, while critical for establishing dose–response relationships and mechanisms of toxicity, significantly exceed typical human environmental exposure. The primary source of population exposure is drinking water, where the maximum contaminant level (MCL) set by the EPA is 3 ug/L (EPA 2025). However, high environmental exposure events, such as seasonal peaks in water, can reach concentrations of up to 30 ug/L for short periods (Holliman et al. 2025).

The discrepancy between animal (mg/kg) doses and the varied human exposure levels (ranging from environmental ug/L to often higher occupational exposures) must be considered when extrapolating risk. Nevertheless, recent epidemiological studies have linked exposure to doses as low as ≥ 0.0804 μg/L to adverse reproductive outcomes in humans, such as an increased risk of preterm birth (Rinsky et al. 2012). Other reports have associated atrazine levels in drinking water (e.g., ≥ 0.644 μg/L) with an elevated prevalence of small for gestational age (SGA) infants (Ochoa-Acuna et al. 2009).

These findings underscore that even those environmental dose exposures, often below or near regulatory limits, are sufficient to cause adverse reproductive effects, thus justifying the urgent need to understand the mechanisms at doses relevant to human exposure.

## Effects of Atrazine on the Hypothalamic-Pituitary–Gonadal Axis

**Fig. 3:**
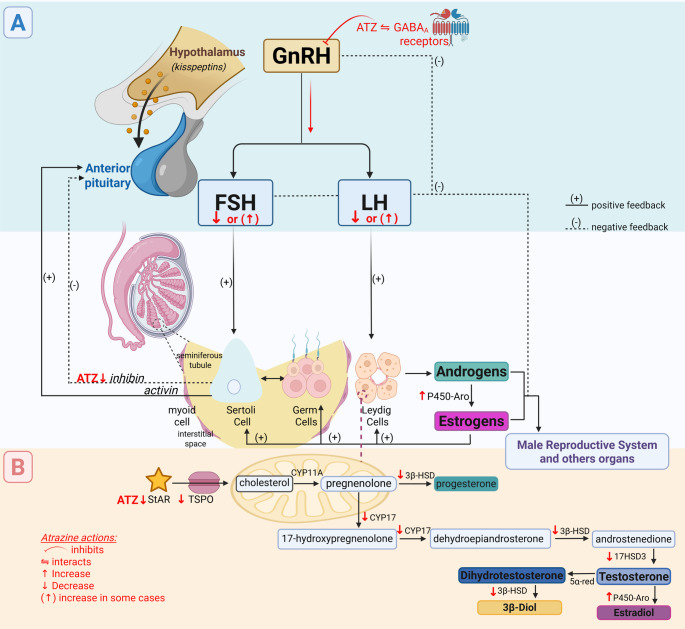
A schematic representation of the effects of atrazine (ATZ) on the Male Rat Hypothalamic-Pituitary-Testicular Axis (A) and testicular steroidogenesis steps (B). Created in https://BioRender.com

The Hypothalamic-Pituitary–Gonadal (HPG) axis (Fig. 3) involves a complex interplay between these three organs and plays a crucial role in regulating sex hormones and reproduction (Vadakkadath Meethal and Atwood 2005). The hypothalamus secretes the gonadotropin-releasing hormone (GnRH) under stimulation by the kisspeptins (de Roux et al. 2003; Seminara et al. 2003; Han et al. 2005). The GnRH stimulates the anterior pituitary gland to release follicle-stimulating hormone (FSH) and luteinizing hormone (LH) (Gnessi et al. 1997). In turn, the FSH and LH act on the testes stimulating spermatogenesis and endocrine functions (Amory and Bremner 2003).

The FSH acts through specific receptors on Sertoli cells, stimulating the production of inhibin (INH-B), activin, and androgen-binding protein (ABP), among other factors. Inhibin exerts a negative feedback effect on FSH release, while activin stimulates its secretion (Gnessi et al. 1997). The ABP is a glycoprotein that binds to androgens, being responsible for their transport and protection against degradation, facilitating their uptake by the male genital tract (Bardin et al. 1981; Jeyaraj et al. 2005). The FSH also controls the proliferation of Sertoli cells during the perinatal period and consequently has been considered the main determinant for the spermatogenic capacity of the adult testis (Heckert and Griswold 1993; França et al., 2016).

The LH stimulates Leydig cells to synthesize testosterone. Together with FSH, testosterone is considered the most important hormone for the control of spermatogenesis, being essential for the initiation and maintenance of this process (Amory and Bremner 2003). Testosterone and its metabolites, dihydrotestosterone (DHT) and estrogens, also act by exerting a negative feedback effect regulating the release of LH and GnRH.

The regulation of gonadotropin secretion by the hypothalamus and pituitary, is similar in both sexes. However, there appear to be fundamental sex differences in the sensitivity of neuroendocrine control to toxicants (Cooper et al. 1999). It is worth mentioning that current data on the effects of atrazine on the HPG axis focuses predominantly on female tissue (Cooper et al. 1995; 1996; 2000; Laws et al. 2000; Davis et al. 2011; Foradori et al. 2009a, 2009b, 2013), thus less is known about the male HPG axis.

Information about the local effects of atrazine in the male hypothalamus and pituitary tissue has been scarce. Some authors reported weight loss in the pituitary after atrazine exposure, concurrent with a decrease in LH levels (Kniewald et al. 2000), suggesting disruptions in the HPG axis.

However, most studies have shown that atrazine indirectly disrupts the HPG axis, leading to changes in circulating LH, FSH, and Inhibin levels (Fig. 3) (Trentacoste et al. 2001; Mokhtari et al. 2011; Feyzi-Dehkhargani et al. 2012; Agdam et al. 2017; Kale et al. 2018; Adedara et al. 2021; Ndufeiya-Kumasi et al. 2022; Ikeji et al. 2023; Rotimi et al., 2023). As shown in Table 2, most studies that measured gonadotrophins levels, observed a reduction in FSH and LH as well as testosterone as part of the disruptive nature of atrazine, irrespective of rat strain (Wistar, Sprague Dawley), developmental stage, duration of the treatment (7, 14, 25, 30, 42, 48, 60 days), and doses of atrazine varying from 40 mg/kg up to 400 mg/kg. Unchanged FSH, LH and testosterone concentration were observed in albino rats (strain not determined) exposed to atrazine in drinking water at 0.01 mg/L to 0.08 mg/L for 12 weeks (Owagboriaye et al 2022), thus further stressing that the dosage used is important to determine the spectrum of effects on these parameters.

**Table 2:** Effects of atrazine on steroidogenesis

References	Dose (atrazine = ATZ)	Route	Hormones/enzymes	Assays	Model
Stoker et al. (2000)	ATZ 12.5, 25, 50 and 100 mg/kg/30d	Gavage	Serum: LH ( =), T ( =), E2 ( =)	RIA	Prepubertal male Wistar rats (21 to 53 PND)
	ATZ 200 mg/kg/30d		Intratesticular: T ↓ Serum: LH ( =), E2 ↑,		
Trentacoste et al. (2001)	ATZ 1, 2.5, 5, 10, 25 and 50 mg/kg/25d	Gavage	Serum: LH ( =), T ( =) Interstitial fluid: T ( =)	RIA	Prepubertal male Sprague–Dawley rats (22 to 47 PND)
	ATZ 100 and 200 mg/kg/25d		Serum: LH ↓, T ↓, Interstitial fluid: T ↓		
Friedmann et al., (2002)	ATZ 50 mg/kg/3 and 27d	Gavage	Serum: T ↓ Testicular: T ↓	RIA	Prepubertal male Sprague–Dawley rats (48 PND)
Rosenberg et al. (2008)	ATZ 1, 10, 50, 75, and 100 mg/kg/7d	Gavage	PND 0—Intratesticular: T ( =) PND 60—Intratesticular: T ↓, Serum: T ↓	RIA	Pre and Pubertal Male Sprague–Dawley rats (PND 0 and PND 60)
Pogrmic et al. (2009)	ATZ 50 mg/kg/27d	Gavage	Serum: LH ( =), T ( =), DHT ( =) Extracellular cAMP ↓ LHR ↓, StAR ↓, TSPO ( =), SF-1 ↓, PDE4B ↓, 3β-HSD ( =), CYP17A1 ↓, 17-βHSD ↓	RIA	Prepubertal male Wistar rats (23 to 50 PND)
	ATZ 200 mg/kg/27d	Gavage	Serum: LH ( =), T ↓, DHT ↓ Extracellular cAMP ↓ LHR ↓, StAR ↓, PDE4B ↓, TSPO ↓, SF-1 ↓, 3β-HSD ( =), CYP 17A1 ↓, 17-βHSD ↓		
Abarikwu et al. (2012b)	ATZ 232 μmol/L/6 h	Cell culture	3β-HSD ↑, StAR ↑ CYP11A1c	ELISA	Prepubertal male Wistar rats (28 PND)
Quignot et al. (2012a)	ATZ 200 mg/kg/14d	Gavage	Testicular: A4 ↓, T ↓, E2 ( =) Serum: A4 ( =), T ( =), E2 ( =) FSH ↑, LH ( =), aromatase activity ↓	LC–MS/MS (T, E2) and ELISA (LH, FSH)	Prepubertal male Sprague–Dawley rats (49 PND)
Song et al. (2014)	ATZ 77 mg/kg/30d	Gavage	Serum: FSH ↑, LH (not evaluated), T ↓, INH-B ↓	ELISA	Prepubertal male Sprague–Dawley rats (28 PND)
	ATZ 154 mg/kg/30d		Serum: FSH ↑, LH ↑, T ↓, INH-B ↓		
Zhu et al. (2021)	ATZ 120 mg/kg/4 weeks	Gavage	Serum: T ↓	N.A	Prepubertal male Sprague–Dawley rats (14 PND)
Rayner et al. (2007)	ATZ 100 mg/kg/4d	Gavage	Serum: T ( =), A4 ( =), E2 ( =)	RIA	Adult male Long-Evans rats
Victor-Costa et al. (2010)	ATZ 50 mg/kg/15d	Gavage	Plasma: T ( =), E2 ( =)	ELISA RIA IHC	Adult male Wistar rats
	ATZ 200 mg/kg/15d		Plasma and testicular: T ↓, E2 ↑ AR protein ( =) Leydig cell 3β-HSD ↓		
	ATZ 200 mg/kg/40d		Plasma: T ↓, E2 ( =) Sertoli cells AR ↓ Leydig cells AR ↑		
	ATZ 300 mg/kg/7d		Plasma: T ↓, E2 ( =) Sertoli and Leydig cells: AR ↓		
Mokhtari et al. (2011)	ATZ 100 mg/kg/14d	Intraperitoneal	Serum: LH ( =), T ↓	RIA	Adult male Wistar rats
	ATZ 200 mg/kg/14d		Serum: LH ↓, T ↓		
	ATZ 400 mg/kg/14d		Serum: FSH ↓, LH ↓, T ↓		
Feyzi-Dehkhargani et al. (2012)	ATZ 100 mg/kg/48d	Gavage	Serum: FSH ↓, LH↓, T ↓, INB ↓	RIA	Adult male Wistar rats
	ATZ 200 mg/kg/48d		Serum: FSH ↓, LH↓, T ↓, INB ↓		
	ATZ 300 mg/kg/48d		Serum: FSH ↓, LH↓, T ↓, INB ↓		
Riffle et al., (2013)	ATZ 200 mg/kg/7d	Gavage	Serum: T ( =), E2 ↑, P4 ↑, CORT ( =)	RIA	Adult male Wistar rats
Abarikwu et al., (2014)	ATZ 120 mg/kg/16d	Gavage	Testicular: 3β-HSD ↓, 17β-HSD ↓	ELISA	Adult male Wistar rats
Riffle et al. (2014)	ATZ 5, 25, 75, and 200 mg/kg/3d	Gavage	Serum: P4 ↑ (25, 75 and 200 mg/kg all dose) Serum: CORT ↑ (all dose groups), LH ( =)	RIA (CORT, P4, T) DELFIA (LH)	Castrated adult male Wistar rats
			Serum: P4 ↑ (25 mg/kg and 200 mg/kg) Serum: CORT ↑ (all dose groups) Serum: LH ↑, T↑ (25 mg/kg)		Intact adult male Wistar rats
Agdam et al. (2017)	ATZ 200 mg/kg/48d	Gavage	Serum: LH ↓, T ↓, IN-B ↓	RIA	Adult male Wistar rats
Martins-Santos et al. (2017)	ATZ 200 mg/kg/7d	Gavage	Aromatase ( =), Leydig cells 3β-HSD ( =)	IHC	Adult male Wistar rats
	ATZ 200 mg/kg/14d		Aromatase ( =), Leydig cells 3β-HSD ↓		
	ATZ 200 mg/kg/40d		Aromatase ↑, Leydig cells 3β-HSD ↓		
	ATZ 200 mg/kg/40d + 75d rec		Aromatase ( =), Leydig cells 3β-HSD ↓		
Aziz et al., (2018)	ATZ 120 mg/Kg/21d	Gavage	Serum: T ↓	RIA	Adult male Albino rats
Kale et al. (2018)	ATZ 300 mg/Kg/7d	Gavage	Serum: FSH ↓, LH ↓, T ↓	RIA	Adult male Wistar rats
Abarikwu et al. (2020)	ATZ 50 mg/kg once a week for 60d	Gavage	3β-HSD ( =) Serum and intratesticular: T ( =)	ELISA	Adult male Wistar rats
Adedara et al. (2021)	ATZ 40 mg/kg/42d	Gavage	Serum: LH ↓, FSH ↓ Serum and intra-testicular: T levels ↓	ELISA	Adult male Wistar rats
Ndufeiya-Kumasi et al. (2022)	ATZ 50 mg/kg/60d	Gavage	Serum: FSH ↓, LH ↓, T ↓	ELISA	Adult male Wistar rats
Owagboriaye et al. (2022)	ATZ 0.01 mg/L for 12 weeks	Drinking water	Serum: LH ( =), FSH ( =), T ( =)	ELISA	Adult male albino rats
	ATZ 0.03 mg/L for 12 weeks		Serum: LH ( =), FSH ( =), T ( =)		
	ATZ 0.04 mg/L for 12 weeks		Serum: LH ( =), FSH ( =), T ( =)		
	ATZ 0.08 mg/L for 12 weeks		Serum: LH ( =), FSH, ( =), T↓		
Ikeji et al. (2023)	ATZ 50 mg/kg/30d	Gavage	Serum and intratesticular: FSH ↓, LH ↓, T ↓	ELISA	Adult male Wistar rats
Rotimi et al., (2023)	ATZ 120 mg/kg/7d	Gavage	Serum: FSH ↓, LH ↓, T ↓	ELISA	Adult male Wistar rats
Hassanin et al. (2024)	ATZ 50 mg/kg/2 months	Gavage	Serum: T ↓	ELISA	Adult male albino rats
Pandey et al. (2021)	ATZ 2 and 10 mg/kg	Subcutaneous (in the F0 dams)	Serum: E2 ↑, AR ↑, ER-α ↑, ER-β ↑, StAR ↑, Aromatase ↑	ELISA	Male Holtzman rats (GD 6 to PND 21)
	ATZ 70 and 100 mg/kg		Serum: T ↓, E2 ↑ AR ↑, ER-α ↑, ER-β ↑, StAR ↑, Aromatase ↑		

↑ increase, ↓ decrease, ( =) Not changed

DELFIA: Dissociation-enhanced lanthanide fluoroimmunoassay, ELISA: Enzyme-linked immunosorbent assay, IHC: Immunohistochemistry, LC–MS/MS: Liquid chromatography with tandem mass spectrometry detection, N.A: Not assessed, RIA: Radioimmunoassays, 17β-HSD: 17β-hydroxysteroid dehydrogenase, 3β-HSD: 3β-hydroxysteroid dehydrogenase, AR: Androgen receptor, cAMP: cyclic adenosine monophosphate, CORT: corticosterone, CYP19A1: aromatase, Cyp17a1: Cytochrome P450 17A1, DHT: dihydrotestosterone, E2: estradiol, ER-α: Estrogen Receptor alpha, ER-β: Estrogen Receptor beta, FSH: follicle-stimulating hormone, GD: Gestation day, GnRH: Gonadotropin-releasing hormone, INH-B: inhibin-B, LC: Leydig cells, LH: Luteinizing hormone, P450scc: cytochrome P450 cholesterol side-chain cleavage enzyme, PDE4B: Phosphodiesterase 4B, SF-1: Steroidogenic Factor 1, StAR: steroidogenic acute regulatory protein, T: testosterone, TSPO: Translocator Protein

A recent systematic review and meta-analysis of hypothalamic-pituitary–testicular axis hormone levels in murine models also concluded that atrazine exposure decreased serum FSH, LH, and testosterone levels, and increased serum estradiol and progesterone levels (Guimaraes-Ervilha et al. 2024). According to these authors, exposure to atrazine in concentrations higher than 100 mg/Kg was the main cause of endocrine disruption, regardless of the exposure time.

Complicating the overall scenario, three studies reported contradictory findings, showing increased FSH and/or LH following atrazine exposure (Riffle et al. 2014; Quignot et al. 2012a; Song et al. 2014). A punctual increase in LH paralleling testosterone levels was found by Riffle et al. (2014), after three days of Wistar rat exposure to 25 mg/Kg atrazine. Song et al. (2014) found increased FSH and LH but decreased testosterone levels in prepubertal Sprague Dawley rats exposed to atrazine at 154 mg/kg for 30 days and proposed that the gonadotropin rise is an indirect effect of atrazine, as a compensatory response to testicular dysfunction and consequent reduction in testosterone and inhibin-B production. The results from Quignot et al. (2012a, bA) were more difficult to conciliate as they found an increase in serum FSH and no change in serum LH, despite observing a decrease in testicular testosterone (with no changes in testicular estrogens) in pubertal Sprague Dawley rats exposed to 200 mg/kg of atrazine for 30 days. Accordingly, atrazine is known to delay puberty in the male rat, impair development and alter the secretion of LH through a direct effect on the central nervous system, so overriding the expected negative feedback response (Stoker et al. 2000).

In order to elucidate the mechanism by which atrazine disrupts the endocrine system and affects the HPG axis, Kniewald et al. (1979) was the first to describe effects of atrazine inhibiting DHT binding to tissue receptor, as shown by in vivo and in vitro experiments, thus demonstrating an inhibitory effect of atrazine on hormone-receptor complex formation. These authors also found inhibition of 5α-reductase and 3α-hydroxysteroid dehydrogenase activity in the male rat anterior pituitary. These enzymes are important, respectively, for conversion of testosterone and its metabolites DHT and 3α-diol, related to the negative feedback effect on LH secretion.

Further on, Shafer et al. (1999) investigated several chlorotriazine herbicides, including atrazine, in the central nervous system and showed that atrazine can interact with GABA_A_ receptors in the brains of male rats, potentially affecting GnRH production. Gamma-aminobutyric acid (GABA) and norepinephrine are known as key regulators of pulsatile release of GnRH from the hypothalamus; therefore, these results gave a clue on how atrazine may interfere with the LH levels and consequently interfere in the testis testosterone production (Shafer et al. 1999).

Feyzi-Dehkhargani et al. (2012) demonstrated that 48 days of exposure to a high dose of atrazine (300 mg/kg/day) resulted in an 85% decrease in serum levels of testosterone, FSH, LH, and INH-B, leading to degeneration of Leydig and Sertoli cells, impaired sperm metabolism, increased oxidative stress, and ultimately, compromised sperm quality. Similar findings were reported by Agdam et al. (2017), who observed reduced serum levels of testosterone, LH, and INH-B after exposure to atrazine (200 mg/kg/day for 48 days). It has been suggested that atrazine exerts its effects primarily by decreasing the synthesis of FSH and LH. In turn, this impairment would lead to Leydig and Sertoli cell dysfunction (Feyzi-Dehkhargani et al. 2012).

In agreement with the impairment in the hormone production, histological damage, exemplified by lesions with areas of fatty infiltration and signs of neuronal degeneration, were found in the hypothalamus of rats treated with atrazine (Adedara et al. 2021; Ikeji et al. 2023). The damages in the brain tissue have been associated with failures in the antioxidative defense (reduced activities of GSH, SOD, CAT, GST, and glutathione peroxidase—GPx, and increased ROS, hydrogen peroxide, MDA, and lipid peroxidation), and inflammation (increased NO, MPO, and interleukin-1β [IL-1β]) (Adedara et al. 2021; Ikeji et al. 2023). Accordingly, Rotimi et al. (2023) also found that the oxide-inflammatory markers were altered in the rat brain, as shown by decreased CAT and SOD activities, and increased MDA, as well as NO, MPO, TNFα, even though they did not detect histological changes in the brain tissue after exposure to atrazine.

From the above, based on the current evidence, the precise mechanism by which atrazine directly affects the HPG axis remains uncertain, suggesting that further research is needed to investigate its direct interactions with this system in males. Atrazine seems to have a more direct impact on steroid hormone production, which subsequently disrupts HPG axis hormones.

## Effects of Atrazine on Testicular Steroidogenesis

Atrazine is a chlorotriazine herbicide, and its specific chemical structure determines its action as an endocrine disruptor. It was demonstrated that atrazine had the greatest inhibitory effect on phosphodiesterase (PDE) when compared to its degradation products, suggesting that the integrity of the triazine structure is essential for its maximum activity (Roberge et al. 2004). On the other hand, atrazine does not strongly mimic estrogen or testosterone through direct, high-affinity binding to their receptors, as evidenced from mechanistic in vitro and in vivo studies (Sanderson et al. 2001; 2002; Roberge et al. 2004; Fan et al. 2007a, b; Victor-Costa et al. 2010). Instead, it appears to exert a strong impact in the disruption of steroidogenesis by altering the expression of key steroidogenic enzymes (Pogrmic et a., 2009; Pogrmic-Majkic et al. 2010; Victor-Costa et al. 2010; Abarikwu et al., 2014; Martins-Santos et al. 2017; Pandey et al. 2021; Guimaraes-Ervilha et al. 2024).

Atrazine may target the testes, affecting steroidogenesis and spermatogenesis (Fig. 3) (Victor-Costa et al. 2010; Martins-Santos et al. 2017; Guimaraes-Ervilha et al. 2024; Hassanin et al. 2024). The structural alterations observed in Leydig cells (discussed in Sect. "Testicular Morphology") align with established evidence demonstrating that atrazine disrupts testosterone levels in rats, regardless of strain, but with influence of exposure time, concentration, and age of animals on the alterations (Guimaraes-Ervilha et al. 2024). Such reduction in testosterone has been a frequent finding, consistently described in serum and/or testicular tissue, particularly at higher doses (Table 2).

Others have raised the possibility that the effects on testosterone levels were secondary to body weight loss induced by atrazine (Trentacoste et al. 2001). However, subsequent research in vitro has evidenced that atrazine has direct effects on Leydig cell androgenesis (Friedman, 2002; Abarikwu et al. 2010, 2012b). It is noteworthy that exposure to atrazine during gestation (50–100 mg/kg) also resulted in decrease in testosterone levels of the offspring at adulthood, although the body weights of these animals were not changed (Rosenberg et al. 2008).

Evidence indicates that the effects of atrazine on testosterone level may be primarily due to its ability to increase the aromatase expression and activity (Wetzel et al. 1994; Crain et al. 1997; Hayes et al. 2002, 2006; Sanderson et al. 2000, 2001, 2006; Heneweer et al. 2004; Laville et al. 2006; Fan et al. 2007a, 2007b; Holloway et al. 2008; Tinfo et al. 2011; Jin et al. 2013; Martins-Santos et al. 2017; Pandey et al. 2021). Aromatase is a key enzyme converting androgens to estrogens, therefore the increase in its activity results in higher estrogen production, but decreased testosterone concentration, as seen after atrazine exposure (Simpson et al. 1994; Sanderson et al. 2000).

Increased plasma estradiol has been reported for rats exposed to atrazine (Stoker et al. 2000, Victor-Costa et al. 2010; Riffle et al., 2013; Pandey et al. 2021). The mechanism of aromatase induction is not fully determined but it appears to involve increase in cyclic adenosine monophosphate (cAMP) levels as a result of inhibition of phosphodiesterase, the enzyme that hydrolyze cAMP to 5′-AMP (Sanderson et al. 2002; Roberge et al. 2004; Kucka et al. 2012). Elevated levels of cAMP ultimately lead to an increase in the aromatase mRNA expression. Fan et al. (2007b) demonstrated that atrazine binds to and activates steroidogenic factor 1 (SF-1) to induce aromatase expression via aromatase promoter II. However, most of the atrazine's effects on aromatase were demonstrated in vitro (Sanderson et al. 2000, 2001, 2002; Heneweer et al. 2004; Betancourt et al. 2006; Laville et al. 2006; Fan et al. 2007a, 2007b; Holloway et al. 2008; Suzawa and Ingraham 2008; Higley et al. 2010; Tinfo et al. 2011; Quignot et al. 2012b; Fa et al. 2013; Caron-Beaudoin et al. 2016).

Supporting these earlier in vitro research, in vivo study by Victor-Costa et al. (2010) revealed elevated estradiol levels alongside decreased testosterone levels in the testes of adult male rats exposed to atrazine. These findings represent the first reported alteration in the estradiol-to-testosterone ratio within rat testes, suggesting that atrazine mechanism of action may indeed involve the induction of estrogen synthesis. Further corroborating with this interpretation, long-term exposure of adult male rats to atrazine also revealed increased aromatase expression in testis, especially in the Leydig cells, as well as in the efferent ductules but not in the ventral prostate following atrazine exposure (Martins-Santos et al. 2017 and 2018). Noteworthy, upon cessation of exposure, testicular aromatase expression returned to control levels (Martins-Santos et al. 2017). Therefore, induction of aromatase expression and activity and the resultant local imbalance between testosterone and estradiol is a plausible explanation for the effects of atrazine in specific reproductive organs.

Whilst aromatase may be an important target for atrazine, other findings suggest that the herbicide's impact on the testicular steroidogenic cascade is more extensive than previously thought. In this regard, evaluation of ex vivo Leydig cells of peripubertal rats demonstrated effects of atrazine on the steroidogenic pathway in Leydig cells, as shown by significant decline in mRNA of several key components involved in the androgen biosynthesis, including luteinizing hormone receptor (LHR), SF-1, phosphodiesterase 4B (Pdeb4), StAR, translocator protein (Tspo), CYP17A1, and 17β-HSD, at both basal and hCG-stimulated conditions (Pogrmic et al. 2009). Further evidencing that atrazine disrupts testosterone production beyond just the final steps of steroidogenesis, reduction in 17β-HSD mRNA and activity and 3β-HSD activity has also been detected in the testicular tissue (Pogrmic et al. 2009; Abarikwu et al., 2014). However, equivalent data on the protein level of these biosynthetic mediators are not available.

Decrease in testis 3β-HSD at protein level was first reported by Victor-Costa et al. (2010), who found Leydig cells weakly stained or negative for this enzyme, whereas there were no changes in adrenal tissue. Further results from Martins-Santos et al. (2017) confirmed decreased 3β-HSD protein in Leydig cell after 15 and 40 days of exposure, especially in testes presenting enlarged or atrophic seminiferous tubules, but not at shorter time effect (7 days). Notably, the enzyme level was not restored after 75 days recovery from the exposure. These authors also reported positivity of testicular macrophages to 3β-HSD, which were significantly increased after a long period of recovery (75 days) from the atrazine exposure, suggesting the involvement of these cells in the regulation of steroidogenesis (Martins-Santos et al. 2017). Accordingly, ultrastructural findings also pointed to changes in interactions between testicular macrophages and Leydig cells after atrazine exposure (Victor Costa et al. 2010). It was interpreted as an attempt to reestablish testicular steroidogenesis, even though despite all efforts, steroidogenesis and the structural damage caused by atrazine were not recovered, thus emphasizing the harmful effects of this herbicide over time.

By using intergenerational investigation on the effects of atrazine (2, 10, 70 and 100 mg/kg), Pandey et al. (2021) also found significant decrease in the serum testosterone and increase in estradiol levels of the F1 offspring rat testis, as well as increase in expression of StAR, aromatase, INSL-3, AR, ERα and ERβ mRNA at all doses tested. The effects on protein level were variable, depending on the dose used, as they found increase in AR at Sertoli and Leydig cells (10, 70 and 100 mg/kg), ERα (70 and 100 mg/kg) and ERβ at Leydig cells and Spermatids/Sertoli cells (100 mg/kg). Lower testosterone and higher estradiol levels were also reported by Jin et al. (2013) in peripubertal rats after gestational exposure to atrazine (50 and 200 mg/kg).

It has been identified that the cyclic adenosine monophosphate/protein kinase A (cAMP/PKA) pathway is an upstream regulator of numerous steroidogenic genes. Effects of atrazine on this pathway appears to be biphasic, as a downregulation of cAMP was found after long term (200 mg/kg for 27 days) exposure to atrazine (Pogrmic et al. 2009), but upregulation after in vitro short-term effect (24 h at 1–50 µM) or in vivo exposure (200 mg/kg for 3 days) of atrazine (Pogrmic-Majkic et al. 2010). Under these latter conditions, Pdeb4, StAR, Tspo and CYP19A1 were not affected, but there was a transient increase in SF-1, CYP17A1, and 17β-HSD, that declined during further treatment (6 days). Later on, by using inhibitors of different signaling pathways, the same group reported that atrazine-induced androgen production depends on the interplay between several signaling pathways such as ERK1/2, ROS, calcium, Protein Kinase C (PKC) and Epidermal Growth Factor Receptor (EGFR), in addition to the cAMP/PKA signaling (Pogrmic-Majkic et al. 2016).

Altogheter, these data highlight that the mechanism of atrazine action in the steroidogenic pathways is still not solved, but that it is certainly much more complicated than first predicted, as atrazine may affect LHR, StAR, Tspo, SF-1, various key enzymes (PDE, CYP17A1, 17β-HSD, 3β-HSD and aromatase) and signaling via (cAMP/PKA, ERK1/2, ROS, PKC and EGFR). What appear consensual in most studies is that this complex mechanism results in a hormonal imbalance, decreasing testosterone but increasing estradiol levels. Considering the estrogen responsive system, although the herbicide does not act directly in the classical estrogen receptors, the effects on non-genomic pathways, such as those driven by the G protein-coupled estrogen receptor (GPER/GPR30), may not be excluded. GPER has been implicated in atrazine-induced cellular effects and cancer cell proliferation (Albanito et al. 2008, 2015), and in activation of EGFR/ERK1/2 pathway, which may also be influenced by atrazine (Pogrmic-Majkic et al. 2016). GPER is present in the testis, efferent ductules and epididymis (Chimento et al. 2010; Lucas et al. 2010; Gomes et al. 2011), however specific studies establishing the effects of atrazine in this protein in male rat reproductive or endocrine tissues are currently lacking, thus deserving future experiments.

## Effects of Atrazine on the Oxidative Pathway

**Fig. 4:**
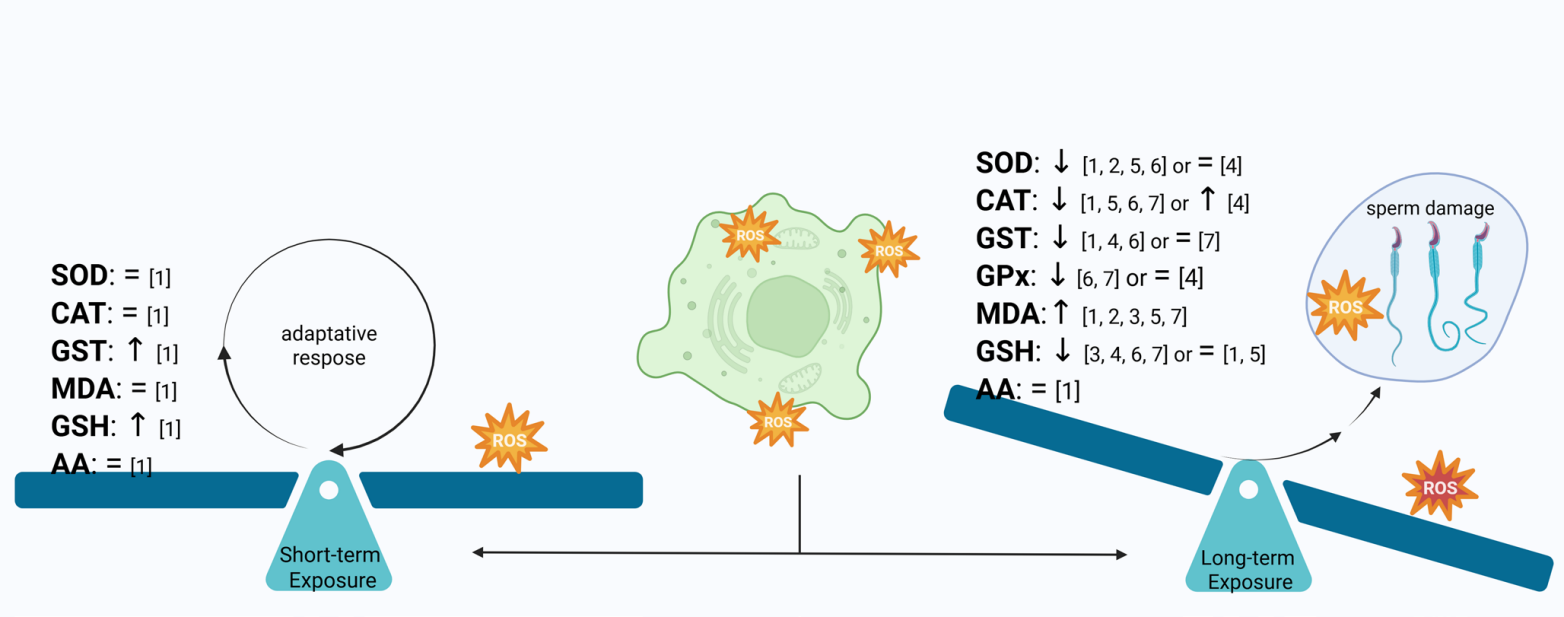
A schematic representation of the effects of atrazine on the oxidative pathway in the male rat (Testis and Epididymis). SOD: Superoxide Dismutase, CAT: Catalase, GST: Glutathione S-transferase, GPx: Glutathione Peroxidase, MDA: Malondialdehyde, GSH: Reduced Glutathione, AA: Ascorbic Acid, ↓Decreased, ↑Increased, = not changed. References: [1] Abarikwu et al., 2010, [2] Abarikwu et al., 2014; [3] Abarikwu et al., 2020, [4] Ikeji et al., 2023, [5] Rotimi et al., 2023, [6] Adedara et al., 2021, [7] Owagboriaye et al., 2022. Created in https://BioRender.com

The mechanisms by which atrazine harms reproduction appear to go beyond the steroidogenic pathway. In this context, there is a body of information showing impacts of atrazine on the oxidative pathway in target organs (Fig. 4) (Abarikwu et al. 2010, 2012b, 2014, 2020, 2023; Feyzi-Dehkhargani et al. 2012; Agdam et al. 2017; Adedara et al. 2021; Ikeji et al. 2023; Owagboriaye et al. 2022; Mgbudom‐Okah et al., 2023; Rotimi et al., 2023; Hassanin et al. 2024).

Reactive oxygen species (ROS) in the oxidative pathway are unavoidable by products of essential cellular processes. Their levels can rise due to various internal and external factors, potentially leading to oxidative stress. Oxidative stress occurs when the body's antioxidant defenses are overwhelmed by an excess of ROS, disrupting cellular functions and damaging macromolecules. If left unchecked, this may result in cell death (Sies 1997; Lushchak and Storey 2021). The cell protection against the damaging effects of ROS is done through antioxidant enzymes, such as SOD, CAT, GST, GPx, glutathione reductase—GR and antioxidants such as GSH and ascorbic acid—AA (Sies 1997; Lushchak 2014). One effect of ROS may be lipid peroxidation, leading to by-products such as the MDA, a mutagenic product that can react with proteins and DNA, leading to cellular damage and dysfunction. Therefore, it is widely used as a biomarker of oxidative stress in various biological samples (Yagi 1987; Gutteridge and Halliwell, 1990).

Atrazine exposure has been shown to trigger a dose-dependent rise in total antioxidant capacity (TAC) and in specific oxidative stress enzymes in the rat testes and epididymis (Abarikwu et al. 2010; Agdam, 2017). At short-term exposure (7 days), atrazine did not significantly affect levels of MDA or the activity of antioxidant enzymes (SOD, CAT) and AA in the testes. However, GSH levels increased, possibly as an adaptive response to oxidative stress. Consequently, GST activity increased, potentially due to increased GSH availability. Thus, atrazine exposure might have generated free radicals in the testes, but the antioxidant system functioned properly, preventing them from causing damage (Abarikwu et al. 2010).

On the other hand, at longer atrazine exposure (16 days), MDA levels increased in both testes and epididymis, suggesting oxidative damage (Abarikwu et al. 2010). SOD and CAT activity decreased in both tissues. GSH and AA levels remained unchanged, whereas GST activity decreased. In these conditions, sperm number and motility decreased, whereas abnormal sperm rate increased, even though there were few morphological changes observed in both the testes and epididymis. Therefore, it appears that long-term exposure overwhelms the antioxidant defenses in both organs, leading to oxidative stress and impaired sperm function (Abarikwu et al. 2010).

Abarikwu et al. (2014) confirmed increased oxidative stress in the testes, as evidenced by higher MDA levels and lower SOD activity, likely contributing to impaired sperm development and function. In another study, the authors reported increased markers of oxidative stress MDA and decreased antioxidant defenses GSH, despite no significant changes in testicular morphology or testosterone levels caused by atrazine (Abarikwu et al. 2020). Other results on atrazine toxicity also showed imbalances in markers of oxidative stress (Ikeji et al. 2023; Rotimi et al., 2023, Adedara et al. 2021). Coincident with increased oxidative stress, atrazine decreased sperm quality, as observed by changes in sperm count, motility, morphology and DNA integrity (Feyzi-Dehkhargani et al. 2012; Agdam et al. 2017; Abarikwu et al. 2020; Ikeji et al. 2023; Rotimi et al., 2023). Similarly, Owagboriaye et al. (2022) reported that atrazine exposure triggered the testes antioxidant defenses, as they found increased levels of ROS, MDA lipid peroxidation, GST and SOD, but decreased GSH, GPx and CAT in the testicular tissue of rats exposed to atrazine at realistic concentrations found in local drinking water (0.08 mg/L). The herbicide induced significant increase in sperm with abnormal tails, thus suggesting that atrazine primarily targets sperm motility, likely by damaging the sperm tail structure.

Considering that atrazine induces toxicity and that this effect is strongly linked to the oxidative stress, there have been many studies addressing different antioxidant compounds that could act as chemopreventive strategies, working to neutralize the damaging effects of atrazine, such as quercetin, fluted pumpkin seeds, curcumin, vitamin E and testosterone, and others natural products (Abarikwu et al., 2014; 2020; 2023; Agdam et al. 2017; Ndufeiya-Kumas et al., 2022; Das et al., 2023). Despite some promising evidence for the role of antioxidants in neutralizing atrazine-induced oxidative stress, they are not conclusive, thus requiring in-depth investigations.

Altogether, growing evidence suggests that atrazine may deplete the antioxidant defense in the testes and epididymis, thus impairing reproductive function, with effects primarily related to sperm damage resulting from oxidative pathway activation.

## Conclusions

**Fig. 5:**
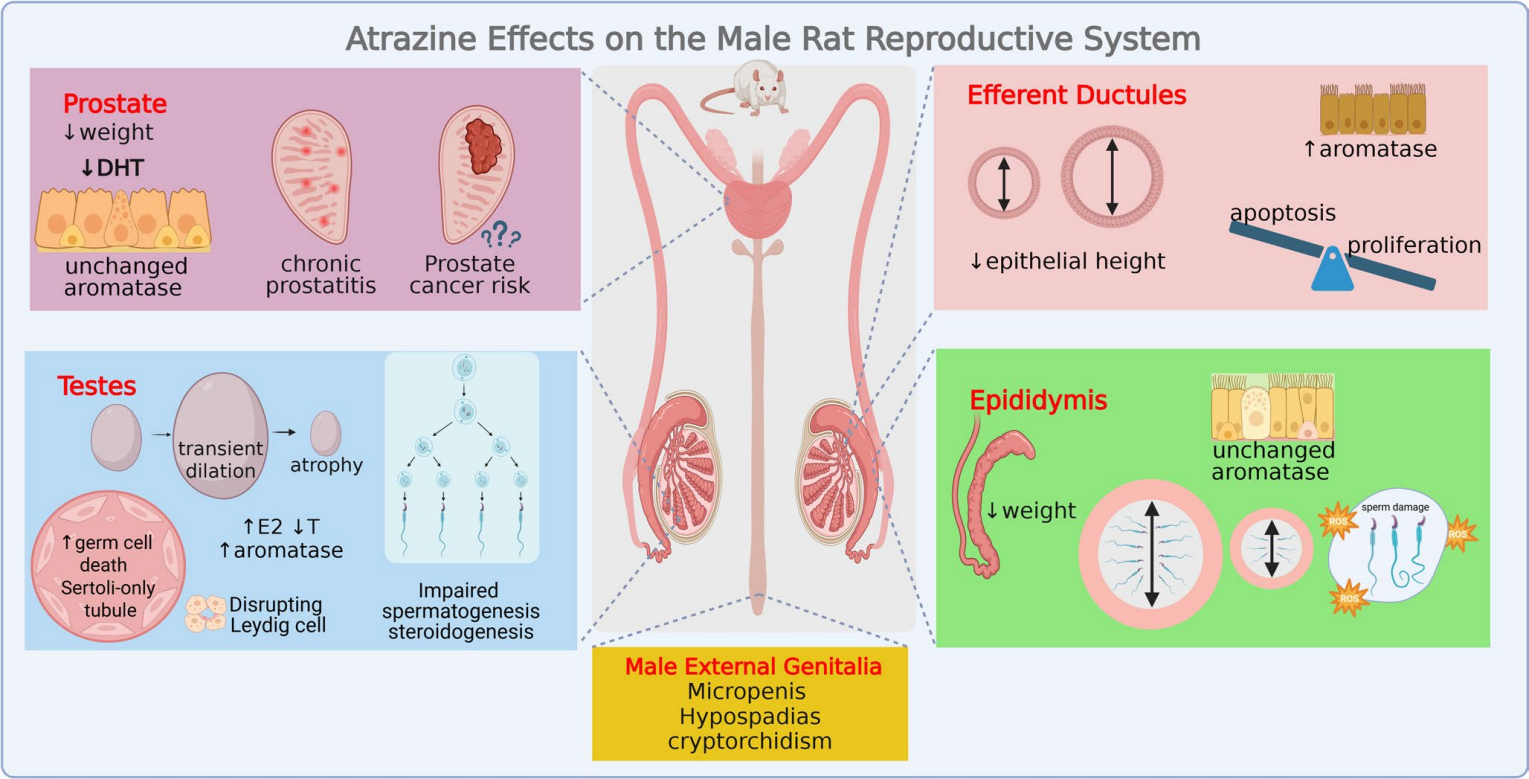
Overview of the effects of atrazine (ATZ) on the male rat reproductive system. Overall information available indicates adverse effects of atrazine on testes, efferent ductules, epididymis, prostate and external genitalia. In summary, the testes may have transient increase in seminiferous tubules luminal dilation and organ weight, followed by atrophy, increased germ cell death and impaired spermatogenesis, resulting in Sertoli-only tubules, aside disrupted Leydig cell function and steroidogenesis leading to decreased testosterone levels but increase in estradiol and aromatase. In the efferent ductules there are increased luminal dilation, reduced epithelial height, increased aromatase expression, and imbalance between proliferation and apoptosis. In the epididymis, it has been observed decrease in organ weight, reduced tubular diameter with increased degeneration, epithelial vacuolation, luminal sperm abnormalities and degeneration, scarcity or absence of sperm, and occasional granulomas leading to obstruction of the epididymal duct and degeneration. The effects on prostates include reduction in DHT and organ weight, unchanged aromatase expression, occurrence of cystic and hyperplastic acini, inflammation, and increased risk of prostate cancer. In the male external genitalia, malformations such as cryptorchidism, reduced penile length and size (micropenis) and hypospadias have been shown. ↓Decrease, ↑Increase, E2 = estradiol, T = Testosterone, DHT = Dihydrotestosterone; the double arrow indicates luminal diameter. Created in https://BioRender.com

We revised the adverse effects of atrazine on the morphophysiology of the male reproductive organs, including the testes, efferent ductules, epididymis, accessory glands and external genitalia, all crucial for male fertility (Fig. 5). It has been shown that atrazine disrupts the hypothalamic-pituitary–gonadal axis, affects key enzymes in steroidogenesis, induces oxidative stress, and consequently disrupts spermatogenesis. Intergenerational effects were also evident, with in utero exposure leading to reproductive abnormalities in offspring, and transgenerational effects have also been observed.

Although relevant progress has been made to understand the effects of atrazine on the male reproduction, significant gaps in our knowledge still exist, especially in the male genital tract downstream the testes. In this sense, the complexity of the atrazine actions in male reproduction is far from being uncovered. For example, although risk of prostate cancer has long been described, few studies focus on animal and pre-clinical investigations on this issue. However, prostate inflammation was frequently found in rodents exposed to atrazine. Chronic prostate inflammation correlates to prostate cancer and benign prostate hyperplasia. Therefore, it cannot be ignored that the incidence of prostate cancer associated with atrazine exposure has increased among men under 60 years old. It is known that the risk of prostate cancer increases with age, so that most cases are diagnosed in men over 65 years. Atrazine was first registered for use in 1958 (WHO 1990), thus just completing 67 years of use worldwide. Considering that prostate cancer is silent and slowly growing, it is worrying and may not be just coincidence that longer follow-up has registered higher incidence of human prostate cancer potentially related to atrazine exposure. Therefore, the atrazine effects on animal and human prostates deserve profound investigations.

Much less is known about the effects of atrazine on external genitalia. Even though disrupting effects, such as cryptorchidism, hypospadias and micropenis, have been found in rodents after prenatal exposure to atrazine, data concerning humans are scarce and controversial. Nevertheless, the incidence of these reproductive disorders has increased in newborns and appears to be related to environmental exposure to endocrine disruptors (Sharpe et al., 2008). Considering that atrazine is an endocrine disruptor globally used, further studies are required to clarify whether atrazine is indeed involved in these developmental disorders as well.

A few recent studies have overcome criticism about the atrazine dosages used in animal models and considered supra-environmental (predominantly 50 to 200 mg/kg), by using more realistic doses found in drink and surface water. Future systematic studies based on such environmentally relevant doses of atrazine encompassing the entire reproductive system and its control by the HPG axis are crucial for a thorough risk assessment. Hopefully, the information compiled in this review, highlighting the current known impacts of atrazine as well as the knowledge gaps, will contribute to future investigations aiming to clarify the atrazine impacts on male health and fertility.

## Data Availability

No datasets were generated or analysed during the current study.
